# Relationship between sensory irritation on skin surface and TRP channel activation

**DOI:** 10.1016/j.jphyss.2026.100096

**Published:** 2026-08-27

**Authors:** Fumitaka Fujita, Masayuki Takaishi, Shizuka Iwashita, Kiyomi Yoshikawa, Maki Sawada, Hironori Shimizu, Moriaki Nagamatsu, Yuko Shiomi, Takeshi Hara, Kenya Ishida, Kunitoshi Uchida, Yoshiro Suzuki, Makoto Tominaga

**Affiliations:** a Mandom Corp., 5-12, Juniken-Cho, Chuoku, Osaka 540-8530, Japan; b Laboratory of Advanced Cosmetic Science, Graduate School of Pharmaceutical Sciences, Osaka University, 1-6, Yamadaoka, Suita, Osaka 565-0871, Japan; c Takasago International Corporation, 1-5-1, Nishiyawata, Hiratsuka, Kanagawa 254-0073, Japan; d Laboratory of Functional Physiology, School of Food and Nutritional Sciences, University of Shizuoka, Suruga-ku, Shizuoka 422-8526, Japan; e Department of Physiology, Iwate Medical University, 1-1-1 Idaidori, Yahaba-cho, Iwate, Shiwa-gun 028-3694, Japan; f Thermal Biology Research Group, Nagoya Advanced Research and Development Center, Nagoya City University, Kawasumi, Mizuho-cho, Mizuho-ku, Nagoya, Aichi 467-8601, Japan

**Keywords:** Sensory irritation, TRPA1, TRPV1, Paraben, Polyol, Arbanol

## Abstract

Mechanisms underlying sensory irritation caused by chemical stimuli on the skin surface remain unknown. Recently, it has been clarified that TRPA1 and TRPV1 are important for sensing nociceptive and temperature stimuli. This study aimed to clarify the mechanism of hTRPA1 and hTRPV1 in sensory irritation on the skin by evaluating the effects of anti-bacterial agents, ethanol, or polyols using electro-physiological methods in HEK293 cells. Parabens (1 mM) activated hTRPA1 in an alkyl chain length-dependent manner. Conversely, high concentrations (>500 mM) of ethanol and polyols activate hTRPV1. The intensities of sensory irritation caused by anti-bacterial agents or polyols correlated with their abilities to activate TRPA1 or TRPV1, respectively. Analogs of known TRPA1 antagonists were screened for their TRPA1 inhibitory activity. Arbanol was identified as a novel TRPA1 inhibitor that can be used to reduce sensory irritation on the skin surface. TRP channels are crucial for sensory irritation on the skin surface.

## Highlights

• Sensory irritations by anti-bacterial agents are correlated with hTRPA1 activities.
• Sensory irritations by ethanol and polyols are correlated with hTRPV1 activities.
• Arbanol is a novel hTRPA1 antagonist with less odor.

## Graphical Abstract

**Figure:**
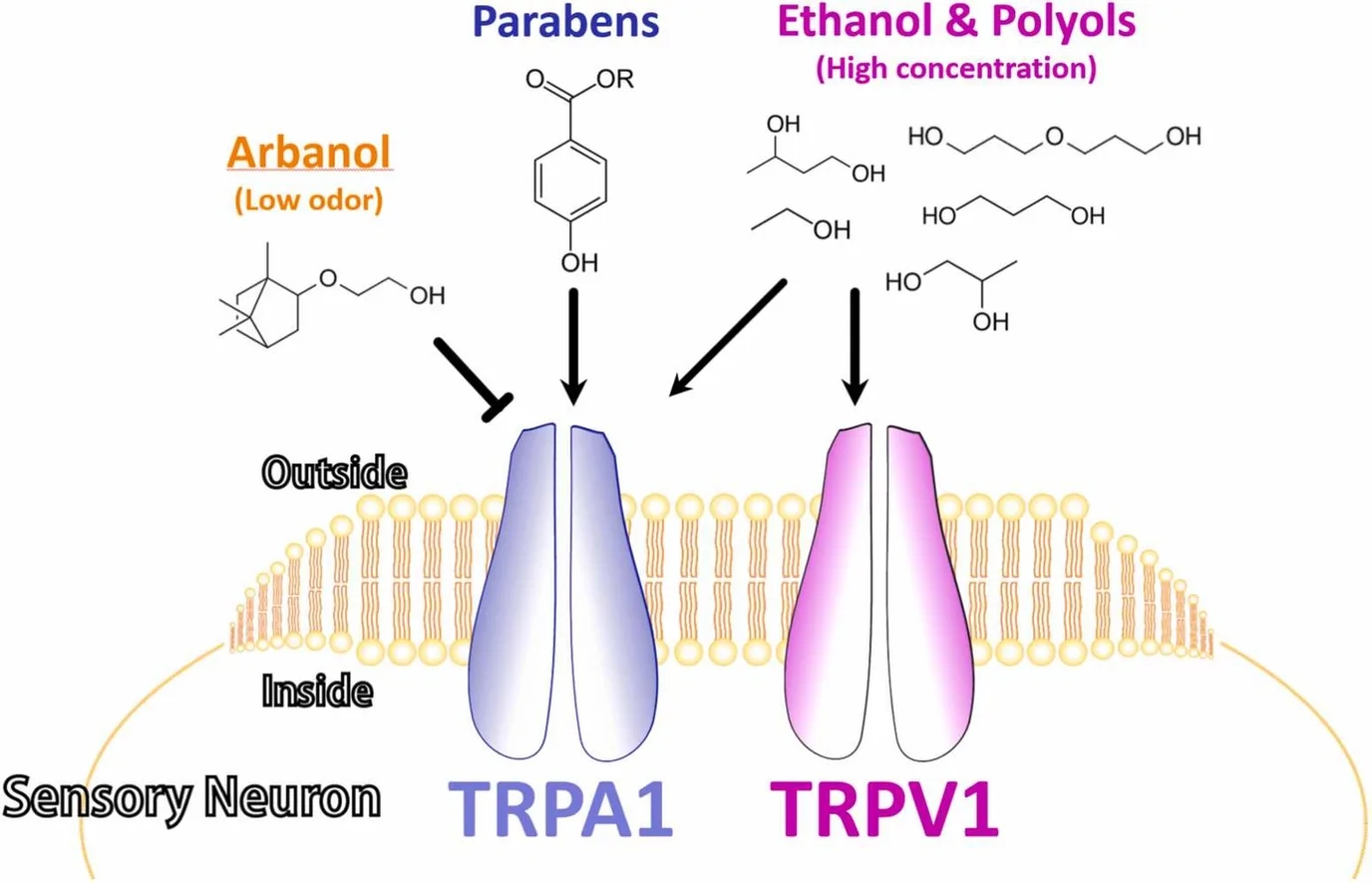

## 1 Introduction

Sensory irritations on the skin, such as stinging, pricking, or burning, are caused by topical cosmetics or medications, although they are not frequent. These irritations caused by skin products are not always accompanied by skin inflammation [1]. Since skin cosmetics are used daily and remain on the skin for extended periods, sensory irritation is unpleasant for consumers. Therefore, this topic has been extensively investigated [2], [3], [4], [5], [6]. Sensitivity to skin cosmetics varies among individuals, necessitating the selection of participants who are sensitive to skin cosmetics for quality evaluation. Sone et al. found that sensitivity to parabens, known to cause stinging [7], and acids varied among participants with skin irritation [8]. Accurate irritation assessment is challenging because participants who are sensitive to specific ingredients must be identified.

The objective evaluation of sensory irritation caused by cosmetics is challenging. However, stinging tests are frequently conducted with skin cosmetics, and sometimes modified skin penetration properties are assessed using non-woven fabrics for a stricter evaluation of sensory irritation [9]. Because stinging tests are labor-intensive and time-consuming, in vitro testing is desirable, although no such tests comparable to human studies have been established.

Sensory irritation levels induced by anti-bacterial agents depend on alkyl-chain length, whereas those caused by high concentrations of ethanol and polyols show diverse results [26]; however, their correlation with TRP channel activity remains unclear. Therefore, we tried to analyze these relationships using human TRP channels activated by anti-bacterial agents, ethanol or polyols, which are well-known irritants causing sensory irritation with cosmetic use. Additionally, we identified nearly odorless TRPA1 inhibitor as a modulator of the skin irritation, which may be a strong candidate for the application in cosmetics.

Transient receptor potential (TRP) channels were first described in Drosophila, in which photoreceptors carrying trp gene mutations exhibited abnormal transient responsiveness to continuous light [10]. TRP channels respond to various stimuli including temperature, nociceptive stimuli, touch, osmolarity, and pheromones [11]. The involvement of TRP channels in nociception has been extensively studied since the cloning of the capsaicin receptor TRPV1 [11], [12]. TRPA1 has also attracted attention for its potential role in nociception [13], [14], [15]. Heterologously expressed TRPA1 channels can be activated by isothiocyanate and thiosulfinate compounds, constituting the pungent ingredients of mustard oil and garlic, respectively [13], [16], [17], [18]. Many cosmetic ingredients or formulation, including paraben, alkali solution, higher alcohol, and hypotonic solution, induce sensory irritation through activation of mice or human TRPA1 [19], [20], [21], [22]. Therefore, we searched for TRPA1 inhibitors among cosmetic ingredients to reduce sensory irritation and identified 1,8-cineole, borneol, 2-methylisoborneol, fenchyl alcohol, and aluminum potassium sulfate [23], [24], [25]. Although these TRPA1 inhibitors are useful for developing non-irritating cosmetics, 1,8-cineole, borneol, 2-methylisoborneol, fenchyl alcohol have inherent unpleasant odors and aluminum potassium sulfate has undissolved characteristics in water at neutral pH that challenges formulators to use them as cosmetic ingredients.

## 2 Methods

### 2.1 Chemicals

Anti-bacterial agents, including parabens, sodium benzoate, and phenoxyethanol and polyols including ethanol, dipropylene glycol (DPG), propylene glycol (PG), 1,3-buthylene glycol (1,3-BG), and glycerin were purchased from Wako Pure Chemical Industries (Osaka Japan). Candidate compounds including 2-((1,7,7-trimethyl-2-bicyclo (2.2.1) heptanyl) oxy) ethanol (arbanol) for TRPA1 inhibitor were obtained from Takasago International Corp. (Tokyo, Japan).

### 2.2 Molecular cloning

Full-length human TRPA1, TRPV1, TRPV2, or TRPM8 cDNAs were obtained from Life Technologies subcloned into pcDNA3.1 vector.

### 2.3 Cell cultures

HEK293T cells were maintained in Dulbecco’s Modified Eagle Medium (Wako Pure Chemical Industries) supplemented with 10% fetal bovine serum (Biowest SAS), 100 U/mL penicillin and 100 µg/mL streptomycin (Thermo Fisher Scientific), and 2 mM L-glutamine (GlutaMAX; Thermo Fisher Scientific), at 37°C in 5% CO_2_ atmosphere. For intracellular Ca^2 +^ imaging, 1 µg of plasmid DNA containing hTRPV1, hTRPA1, hTRPV2, and hTRPM8 in pcDNA3.1 was transfected into HEK293T cells using Lipofectamine Plus Reagent (Thermo Fisher Scientific) in OPTI-MEM (Thermo Fisher Scientific). After a 3–4 h incubation, the cells were reseeded onto coverslips and incubated overnight at 37°C in 5% CO_2_.

### 2.4 Ca^2+^ imaging

Ca^2+^ imaging was performed one day after transfection of hTRPV1, hTRPA1, hTRPV2, or hTRPM8 cDNAs into HEK293T cells. Each cell was mounted onto coverslips in an open chamber and superfused with a standard bath solution (140 mM NaCl, 5 mM KCl, 2 mM MgCl_2_, 2 mM CaCl_2_, 10 mM HEPES, and 10 mM glucose; pH 7.4). Free cytosolic Ca^2+^ concentrations in HEK293T cells were measured by dual-wavelength microfluorometry using a Fura-2 radiometric indicator (Thermo Fisher Scientific) (excitation; 340/380 nm, emission; 510 nm). The Fura-2 ratio was calculated using the IPlab imaging processing system (Scanalytics Inc.). All experiments were performed using Nikon ECLIPS Ti microscope.

### 2.5 Electro-physiological studies

Whole-cell patch-clamp recordings were performed one day after transfecting hTRPV1, hTRPA1, hTRPV2, or hTRPM8 cDNAs into HEK293T cells. The standard bath solution was the same as that used in the Ca^2+^ imaging experiments. The pipette solution contained 140 mM KCl, 5 mM EGTA, and 10 mM HEPES, pH 7.4 (with KOH). Data from whole-cell voltage-clamp recordings were sampled at 10 kHz and filtered at 5 kHz for analysis using Axon 1550B amplifier with pCLAMP software (Axon Instruments). The membrane potential was clamped at −60 mV under all conditions. All experiments were performed at room temperature (24–26 °C). Voltage ramp-pulses from −100 mV to +100 mV (500 ms) were applied every 5 s to generate a current-voltage curve. All experiments were performed using Nikon ECLIPS Ti Microscope.

### 2.6 Participants

Ethical approval (No.101–7, 102–34) and informed consent were obtained prior to the analysis. The Japanese participants were in their 20 s and 30 s. To evaluate sensory irritation, male participants who were sensitive to menthol were selected (mean age: 32.5 ± 4.5 years). To evaluate the odor levels, male and female participants who were sensitive to odors were chosen (mean age: 29.4 ± 4.5 years).

### 2.7 Olfactory evaluation

TRPA1 inhibitor solutions (1%) in 50% ethanol were used as odor samples. Odor was presented using smelling strips and evaluated on a six-point intensity scale (Table 1).

**Table 1:** Criteria for odor scoring.

Score	Scoring criteria
5	Extremely strong
4	Strong
3	Distinct
2	Weak (Recognition threshold)
1	Very weak (Detection threshold)
0	No odor

### 2.8 Sensory irritation tests

This study was conducted at 21–23°C and 45–55% relative humidity. The skin was cleaned with a wet towel and acclimatized for 10 min before testing. Blind randomized half-region (left vs. right) trials were performed with two different samples applied to the neck. The amount of base applied was 500 μL. The participants evaluated prickly, stinging, burning and itching sensations 1, 3, 5, 7, 9, 11, 13, 15, 17 and 20 min after compounds were applied (Table 2). We used scores for stinging and burning sensations induced by TRPA1 activation. Scores for 2 min before and after the period when the application of menthol alone caused maximum irritation were chosen for analysis. The tests were conducted 12 times with nine men. Datasets from men were obtained at 5–9 min and 7–11 min after the application. Ethical approval of experiments involving human skin were obtained in Mandom Corporation (No.101–7, 102–34).

**Table 2:** Criteria for sensitivity scoring.

Seosory expression	Score	Scoring criteria
Stinging pain	5	Unbearable intense sensation
Painful prickly sensation	4	⇳
Burning sensation	3	Distinct sensation
Itching	2	⇳
Slightly unusual	1	Obscure sensation
	0	No sensation

### 2.9 Data analysis

Data in all figures were shown as the mean ± standard error of the mean (SEM), and p < 0.05 were considered significant. The statistical significance of the effects of the ingredients was evaluated using Student’s *t*-test or Dunnett’s test (electrophysiological experiments), Steel-Dwass test (olfactory evaluations), and Mann-Whitney *U* test (sensory irritation tests). Dose-dependent curves were fit using Hill equation.

## 3 Results

### 3.1 Anti-bacterial agents on TRPA1

**Fig. 1:**
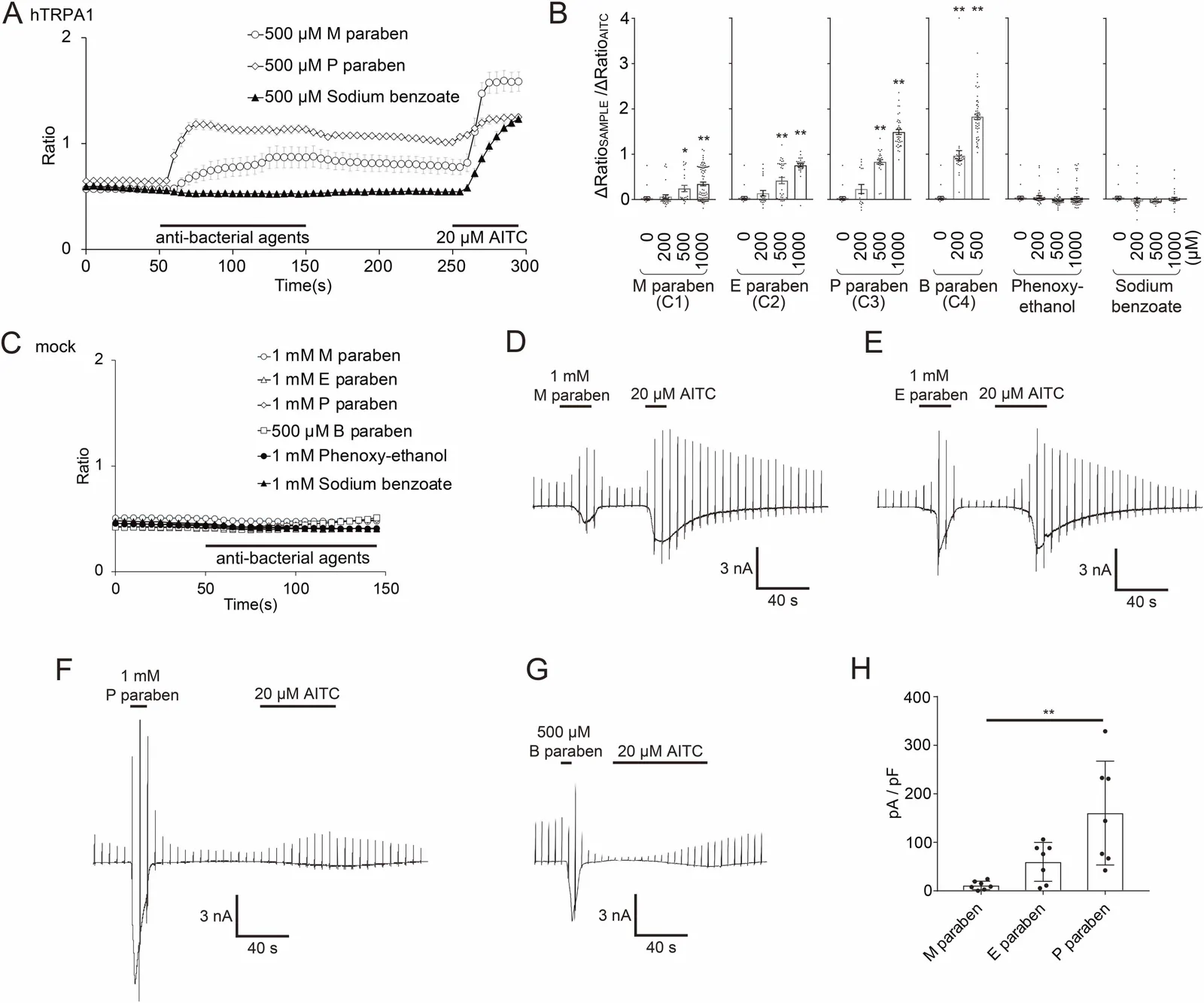
Increases in cytosolic calcium concentrations and activation currents by anti-bacterial agents in HEK293 cells expressing hTRPA1.(A) Changes in [Ca^2+^]_i_ induced by anti-bacterial agents; 500 μM of methyl- (n = 23), ethyl (n = 35) and propyl-paraben (n = 27), or sodium benzoate (n = 22), in the cells expressing hTRPA1. **(B)** Comparison of changes in [Ca^2+^]_i_ induced by anti-bacterial agents in the cells expressing hTRPA1. Methyl (200 μM; n = 34, 500 μM; n = 23, 1 mM; n = 81), ethyl (200 μM; n = 23, 500 μM; n = 35, 1 mM; n = 31), propyl (200 μM; n = 18, 500 μM; n = 27, 1 mM; n = 39), butyl (200 μM; n = 31, 500 μM; n = 53) paraben, phenoxy-ethanol (200 μM; n = 45, 500 μM; n = 59, 1 mM; n = 89), and sodium benzoate (200 μM; n = 25, 500 μM; n = 22, 1 mM; n = 38). Data are shown as a delta ratios normalized by the ratios caused by AITC. * p < 0.05, ** p < 0.01 vs negative control. (C) Changes in [Ca^2+^]_i_ by 1 mM of methyl- (n = 51), ethyl (n = 17) and propyl-paraben (n = 27), 500 mM of butyl paraben (n = 28), 1 mM of phenoxy-ethanol (n = 16), or 1 mM of sodium benzoate (n = 30) in the mock transfected cells. (D-G) Representative traces of the TRPA1-mediated currents activated by 1 mM methyl (D), ethyl (E), propyl (F) paraben or 500 mM of butyl paraben (G) in the presence of extracellular Ca^2+^. (H) Comparison of the densities of hTRPA1 currents activated by methyl (n = 7), ethyl (n = 7), or propyl paraben (n = 7). ** p < 0.01 vs methyl paraben.

We examined the abilities of anti-bacterial agents to activate human TRPA1 using Ca^2+^-imaging. In the anti-bacterial agents, 1 mM of methyl-, ethyl-, propyl-, and butyl-paraben caused [Ca^2+^]_i_ increases in cells expressing hTRPA1, but not in the mock-transfected cells (Fig. 1A, B, C). Moreover, the [Ca^2+^]_i_ increases by parabens showed an alkyl-chain length-dependent manner. However, phenoxyethanol and sodium benzoate did not cause [Ca^2+^]_i_ increases (Fig. 1A, C).

On the other hand, 1 mM parabens, phenoxyethanol, or sodium benzoate did not cause [Ca^2+^]_i_ increases in the cells expressing other TRP channels expressed in sensory neurons (Additional Fig. 1A-C), while small [Ca^2+^]_i_ increases were observed in the cells expressing hTRPV1 or hTRPV2 (Additional Fig. 1A, C). These results indicate that paraben-induced [Ca^2+^]_i_ increases are mainly mediated by TRPA1 in humans, which is similar to a previous report on mouse TRPA1 [18].　Among the anti-bacterial agents, 1 mM methyl-, ethyl-, propyl-, and butyl-paraben induced current activation of hTRPA1 in the patch-clamp experiments (Fig. 1D-G). Moreover, TRPA1 activation by parabens was alkyl-chain length-dependent manner (Fig. 1H) similar to the Ca^2+^-imaging experiments. Furthermore, the small hTRPA1 currents observed after the application of propyl- or butyl-paraben at 20 mM suggest significant desensitization of hTRPA1 after strong activation (Fig. 1F, G).

### 3.2 Ethanol and polyols on TRPV1

**Fig. 2:**
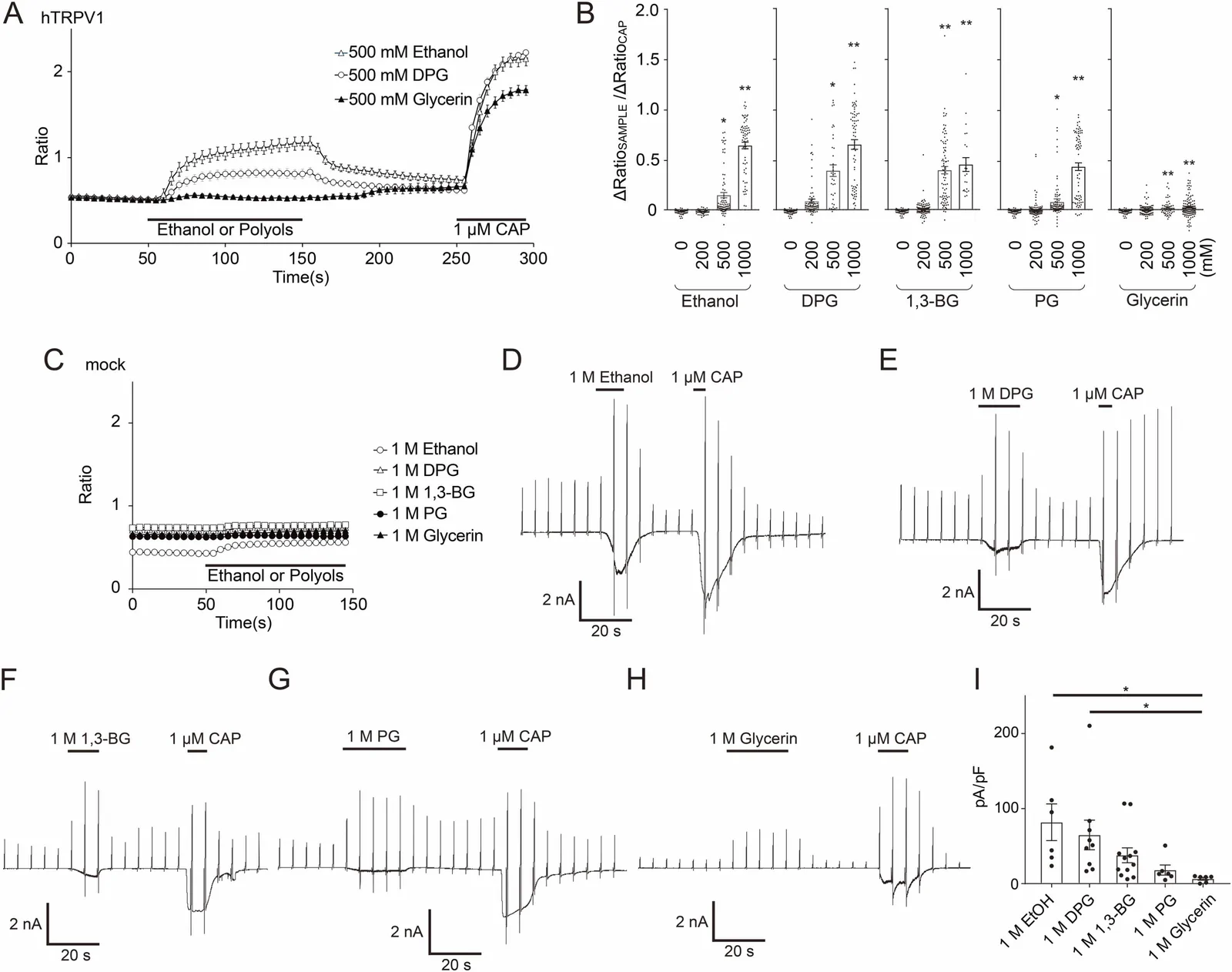
Increased cytosolic calcium concentration by ethanol or polyols in HEK293 cells expressing hTRPV1. (A) Cahnges in [Ca^2+^]_i_ induced by 500 mM of ethanol (n = 82), DPG (n = 37) or glycerin (n = 52) in the cells expressing hTRPV1. **(B)** Comparison of changes in [Ca^2+^]_i_ induced by ethanol or polyols in the cells expressing hTRPV1. Ethanol (200 mM; n = 6 9, 500 mM; n = 82, 1 M; n = 71), DPG (200 mM; n = 75, 500 mM; n = 37, 1 M; n = 70), 1,3-BG (200 mM; n = 116, 500 mM; n = 83, 1 M; n = 22), PG (200 mM; n = 150, 500 mM; n = 80, 1 M; n = 75), and glycerin (200 mM; n = 146, 500 mM; n = 52, 1 M; n = 119). Data are shown as delta ratios normalized by the ratios caused by capsaicin. * p < 0.05, ** p < 0.01 vs negative control. **(C)** Changes in [Ca^2+^]_i_ induced by 1 M ethanol (n = 41), DPG (n = 61), 1,3-BG (n = 41), PG (n = 46) and glycerin (n = 24) in the mock-transfected cells. **(D-H)** Representative traces of theTRPV1-mediated currents activated by 1 M ethanol **(D)**, DPG **(E)**, 1,3-BG **(F)**, PG **(G)**, or glycerin **(H)** in the presence of extracellular Ca^2+^. (I) Comparison of the densities of hTRPA1 currents activated by 1 M of ethanol (n = 6), DPG (n = 9), 1,3-BG (n = 12), PG (n = 6) or glycerin (n = 6). * p < 0.05 vs glycerin.

500 mM ethanol, DPG, 1,3-BG, or PG, but not glycerin, caused significant and large [Ca^2+^]_i_ increases in HEK293T cells expressing hTRPV1 (Fig. 2A, C). In the mock-transfected cells, no [Ca^2+^]_i_ increase was observed for any stimulus except for 1 M ethanol (Fig. 2C). However, this increase by ethanol was very small compared to that in cells expressing hTRPV1 (Fig. 2A, C). Higher concentration (500 mM) of ethanol- or polyol-induced [Ca^2+^]_i_ increases were approximately 50% of those caused by 1 μM capsaicin (Fig. 2A), suggesting that ethanol and polyols have modest abilities to activate hTRPV1. To determine whether ethanol or polyol-induced [Ca^2+^]_i_ increases are specific to TRPV1, we examined the effects of 1 M ethanol or polyols on [Ca^2+^]_i_ in HEK293T cells expressing other TRP channels (TRPA1, TRPV2, or TRPM8) which are expressed in sensory neurons and involved in nociception or pain relief.

Ethanol and polyols caused small [Ca^2+^]_i_ increases in HEK293T cells expressing hTRPA1 or hTRPV2, but not in cells expressing hTRPM8 (Additional Fig. 2A-C), suggesting the specific action of ethanol and polyols on hTRPV1, hTRPA1, and hTRPV2 channels. To examine whether ethanol or polyols activate hTRPV1, we performed patch-clamp experiments using HEK293T cells expressing hTRPV1. 1 M ethanol or DPG evoked significant inward currents at −60 mV with apparent outward rectification (Fig. 2D, E). In contrast, 1 M 1,3-BG, PG, or glycerin evoked only small inward currents (Fig. 2F-H). hTRPV1-activating abilities of 1 M of ethanol or DPG were significantly larger than that of glycerin (Fig. 2I).

### 3.3 Correlation between sensory irritation and TRP channels’ activities

**Fig. 3:**
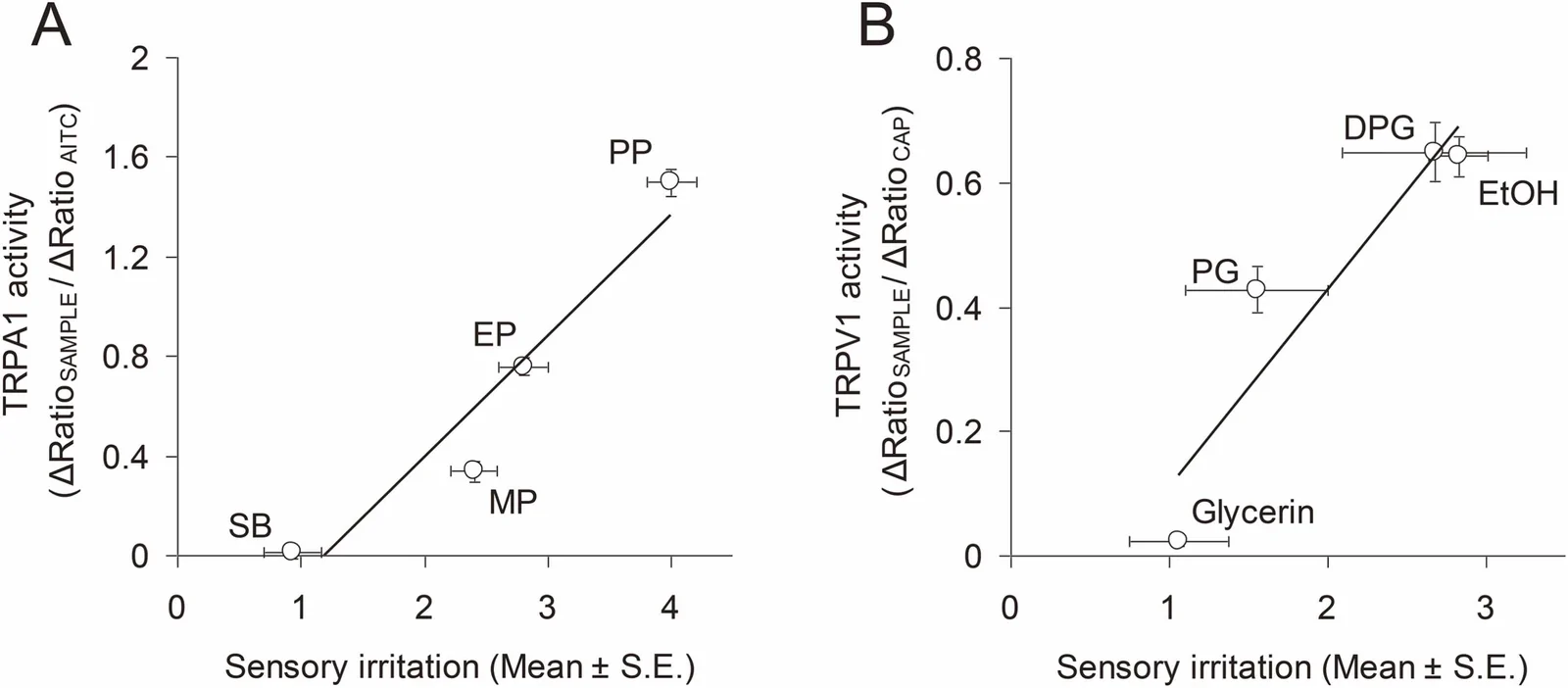
Correlations between TRPA1 or TRPV1 activities induced by anti-bacterial agents or polyols and sensory irritation scores. (A) Correlation between TRPA1 activities induced by antibacterial agents and sensory irritation induced by them. SB: sodium benzoate, MP: methyl-paraben, EP: ethyl-paraben, PP: propyl-paraben. **(B)** Correlation between TRPV1 activities induced by ethanol or polyols and sensory irritation induced by them. PG: propylene glycol, DPG: dipropylene glycol.

TRPA1 activities against anti-bacterial agents were analyzed in the correlation with sensor irritation levels [26]. The relationship between TRPA1 activities and sensory irritation levels measured by a previously established method showed positive correlation (R=0.96, Fig. 3A). Moreover, TRPV1 activities and sensory irritation levels were positively correlated, too (R=0.93, Fig. 3B). These results strongly suggested hTRPA1 and hTRPV1 are useful makers or estimation of sensory irritation induced by cosmetic ingredients or formulations.

### 3.4 Arbanol as a novel TRPA1 inhibitor

**Fig. 4:**
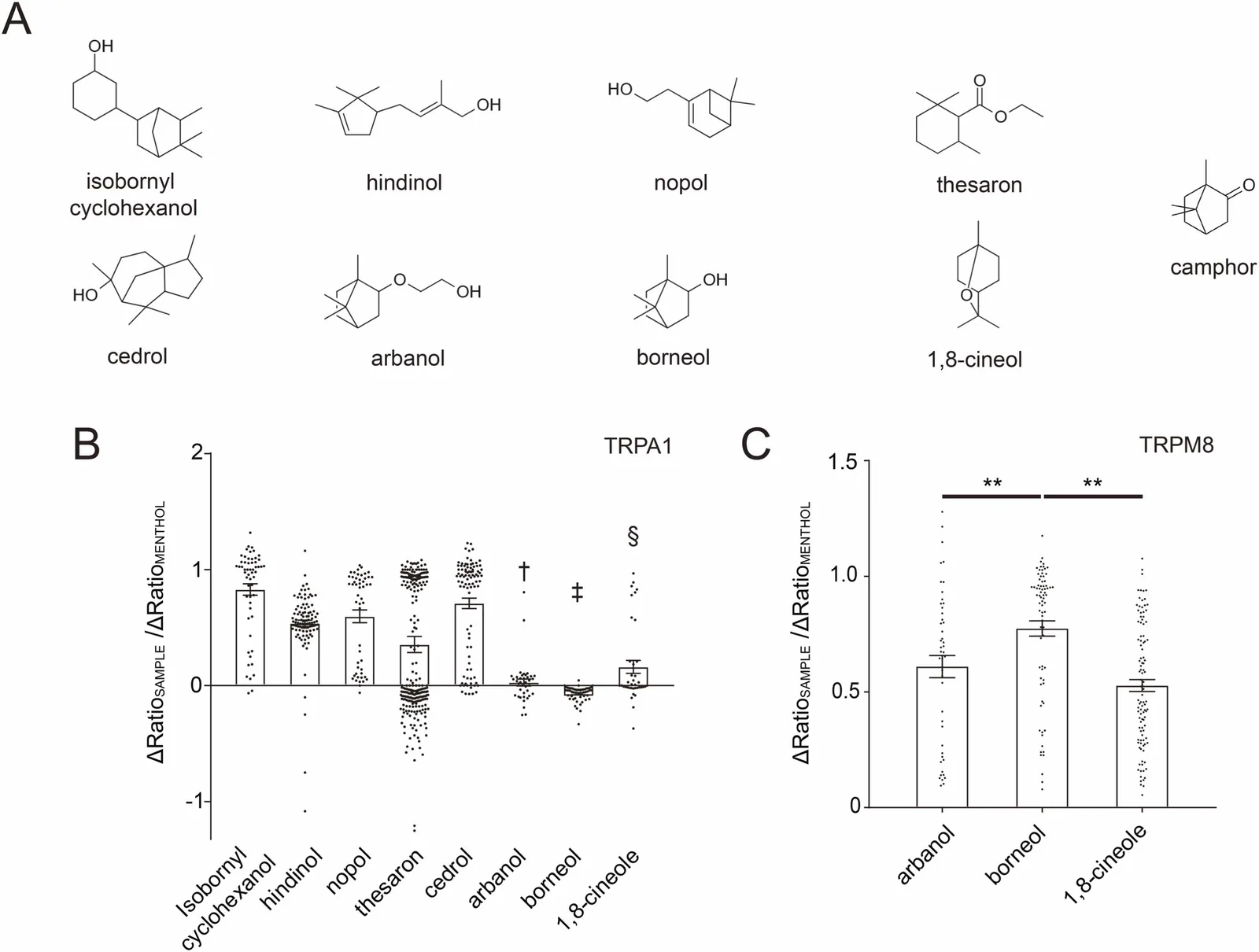
**Effects of arbanol and analogs of TRPA1 inhibitors on TRPA1 or TRPM8 activities. (A)** Molecular structures of candidate chemicals with camphor as a reference. **(B)** Comparison of changes in [Ca^2+^]_i_ by 1 mM of candidates in HEK293 cells expressing hTRPA1 (isobornyl cohexanol; n = 56, hindinol; n = 101, nopol; n = 51, thesaron; n = 274, cedrol; n = 92, arbanol; n = 38, borneol; n = 52, 1,8-cineole; n = 38). Data are shown as delta ratios normalized by the ratios caused by menthol. † p < 0.01 vs isobornyl cohexanol, p < 0.05 vs hindinol, p < 0.05 vs nopol, and p < 0.01 vs cedrol. ‡ p < 0.01 vs isobornyl cohexanol, p < 0.01 vs hindinol, p < 0.01 vs nopol, p < 0.01 vs thesaron, and p < 0.01 vs cedrol. § p < 0.01 vs isobornyl cohexanol, and p < 0.01 vs cedrol. **(C)** Comparison of [Ca^2+^]_i_ increases by 1 mM of arbanol (n = 48), borneol (n = 76) or 1,8-cineole (n = 106) in HEK293 cells expressing hTRPM8. Data are shown as delta ratios normalized by the ratios caused by menthol. ** p < 0.01.

We previously reported that 1,8-cineole and borneol inhibit hTRPA1 [23], [24]. However, they have a specific unpleasant odor. We hypothesized that hTRPA1 inhibitors without odor could be used as ingredients to control sensory irritation. Following our previous studies [23], [24], we screened the effects of the candidate inhibitors (Fig. 4A) selected from analogs of the natural hTRPA1 antagonists; camphor and 1,8-cineole, on hTRPA1 activity using Ca^2+^-imaging in HEK293T cells. Arbanol did not change menthol-induced [Ca^2+^]_i_ when being co-applied with menthol which activates hTRPA1, like 1,8-cineole or borneol (Fig. 4B), indicating that arbanol does not have a TRPA1-activating ability. Moreover, arbanol caused an increase in [Ca^2+^]_i_ and significant currents in cells expressing hTRPM8 (Fig. 4C and Additional Fig. 3A, B), indicating that arbanol causes a cooling sensation. The effects of arbanol on hTRPA1 and hTRPM8 were similar to those of 1,8-cineole and borneol, which are reported as TRPA1 inhibitors [23], [24], suggesting that arbanol may act as a TRPA1 inhibitor.

**Fig. 5:**
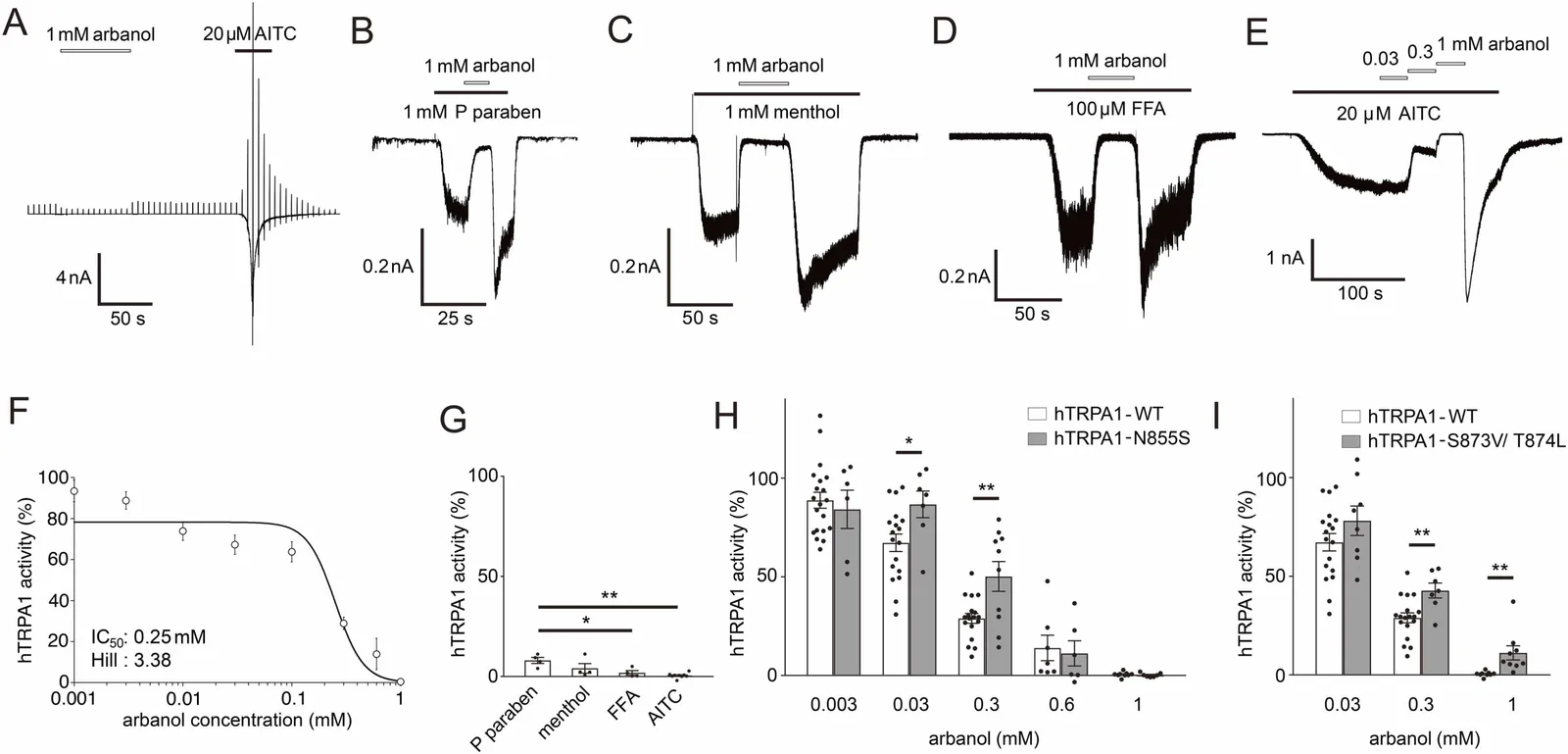
Inhibitory effect of arbanol on hTRPA1-mediated currents in the cells expressing hTRPA1. (A) A representative current trace with1 mM arbanol. **(B-E)** Representative traces of hTRPA1-mediated currents acctivated by 1 mM of propyl paraben **(B)**, 1 mM of menthol **(C)**, 100 μM of FFA **(D)** or 20 μM of AITC **(E)** inhibited by arbanol. **(F)** Dose-dependent inhibition of 20 μM AITC-evoked hTRPA1 current by arbanol (n = 6–19). The half-maximal inhibitory concentration (IC_50_) and Hill’s coefficient values were 0.25 mM and 3.38, respectively. **(G)** Comparison of hTRPA1 currents by 1 mM of propyl paraben, 1 mM of menthol, 100 μM of FFA or 20 μM of AITC with 1 mM arbanol. * p < 0.05, ** p < 0.01. **(H)** Comparison of hTRPA1 activities normalized to the values without arbanol in the cells expressing wild type (hTRPA1-WT, 0.003 mM; n = 19, 0.03 mM; n = 17, 0.3 mM; n = 18, 0.6 mM; n = 6, 1 mM; n = 11) and mutants (N855S, 0.003 mM; n = 6, 0.03 mM; n = 7, 0.3 mM; n = 10, 0.6 mM; n = 6, 1 mM; n = 7). * p < 0.05, ** p < 0.01 vs WT. **(I)** Comparison of hTRPA1 activities normalized to the values without arbanol in the cells expressing wild type (hTRPA1-WT, 0.03 mM; n = 17, 0.3 mM; n = 18, 1 mM; n = 11) and mutants (S873V/ T874L, 0.03 mM; n = 8, 0.3 mM; n = 7, 1 mM; n = 9). ** p < 0.01 vs WT.

To confirm the effects of arbanol on hTRPA1, we conducted patch-clamp experiments in the cells expressing hTRPA1. Arbanol (1 mM) did not evoked hTRPA1-mediaed currents by itself (Fig. 5A), which was in line with our Ca^2+^-imaging results. Moreover, propyl paraben-, menthol-, FFA-, or AITC-evoked hTRPA1 currents were inhibited by 1 mM arbanol (Fig. 5 B-E). Among them, we analyzed the details of AITC-evoked hTRPA1 currents and found that arbanol inhibits hTRPA1 activity in a dose-dependent manner with a half-maximal inhibitory concentration (IC_50_) of 0.25 ± 0.04 mM (Fig. 5E, F). These IC_50_ values were similar to those of borneol (0.20 ± 0.06 mM) [23], and lower than those of 1,8-cineole (3.43 ± 0.58 mM) [22]. Although the inhibition of 1 mM propyl paraben-evoked hTRPA1 currents by arbanol was weaker than that of AITC- or FFA-evoked currents, arbanol clearly inhibited those hTRPA-mediated currents by over 90% (Fig. 5G). These results suggest that arbanol is an effective TRPA1 inhibitor. Furthermore, we estimated the possible binding sites of arbanol using two types of TRPA1 mutants (N855S and S873V/T874L), in which asparagine 855 was reported to be involved in AITC-evoked activation [27], and serine 873 and threonine 874 were reported to be involved in menthol-evoked activation [28]. Similar to borneol, the inhibitory effects of arbanol on hTRPA1 were partially but significantly reduced in cells expressing TRPA1-N855S or TRPA1-S873V/T874L, suggesting that these three residues are involved in the inhibitory effect of arbanol (Fig. 5H, I).

### 3.5 Characteristics of arbanol on human participants, smell, and analgesic effect

**Fig. 6:**
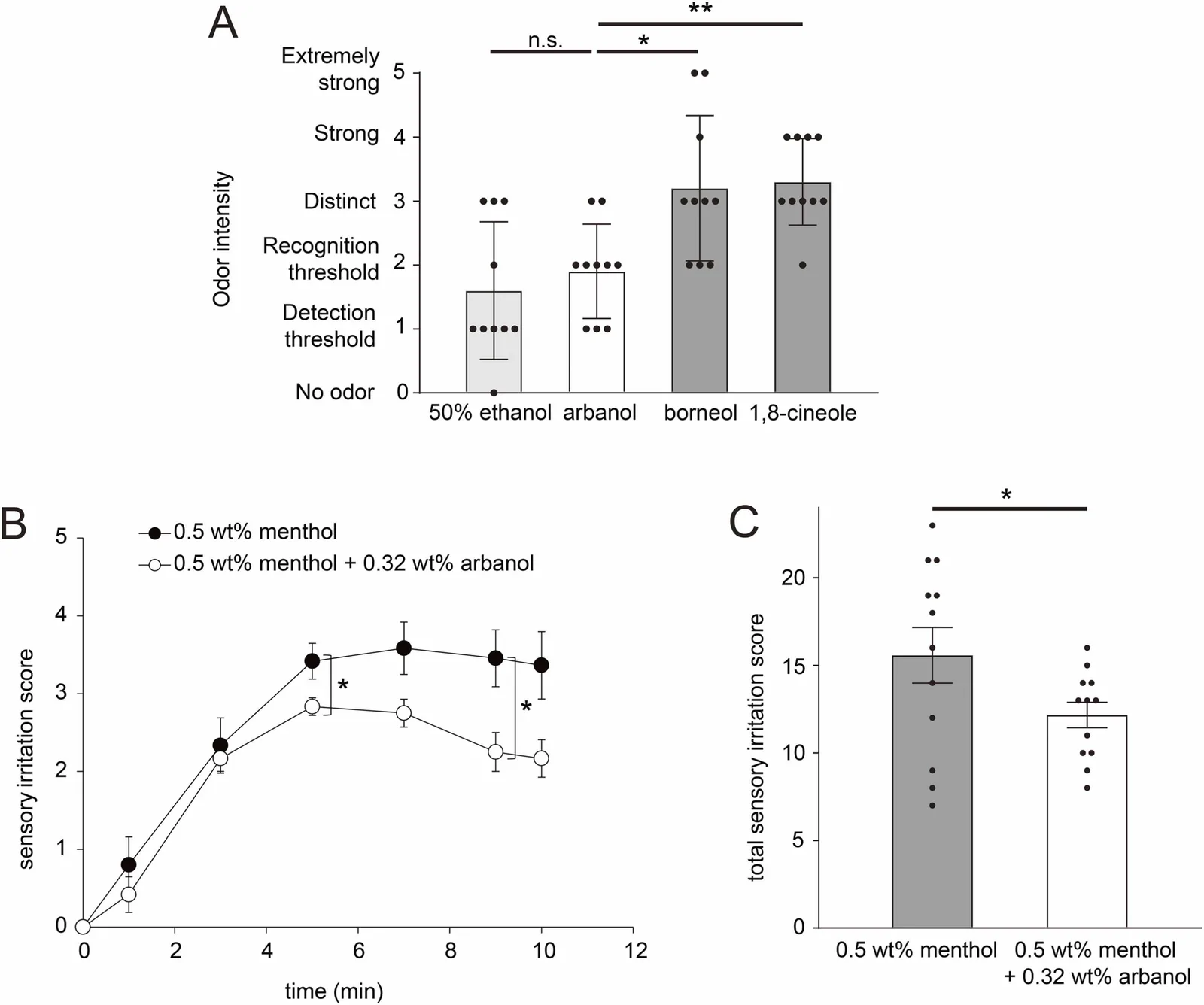
Effects of arbanol in the evaluation of odor levels or inhibitory effect for sensory irritation evoked by menthol on human participants. (A) Oder levels of arbanol among known TRPA1 inhibitors (n = 10). **(B)** Sensory irritation caused by 0.50 (wt%) menthol was significantly inhibited by concomitant application of 0.32 (wt%) arbanol 5 or 9 min after application (n = 10–12). **(C)** Total score of sensory irritation by menthol was significantly inhibited by arbanol (n = 12). Statistical significance was evaluated using Wilcoxon signed-rank test. * p < 0.05.

We evaluated the odor intensities of arbanol, borneol, 1,8-cineole, and a solvent without compounds (50% ethanol). The odor intensity score of arbanol was significantly lower than those of borneol or 1,8-cineole (Fig. 6A). Sensory irritation tests in human participants confirmed the arbanol effect on hTRPA1 inhibition against menthol-evoked activation in vivo. The concomitant application of 0.5% menthol with arbanol significantly reduced the sensory irritation scores in sensitive participants (Fig. 6B, C). These results show that arbanol can reduce sensory irritation when applied concomitantly with menthol.

## 4 Discussion

There have been no detailed reports on in vitro tests for sensory irritation on the skin surface. This study confirmed that hTRPA1 activities by anti-bacterial agents or hTRPV1 activities by high concentrations of ethanol or polyols show positive correlation with sensory irritation levels in human subjects. Arbanol, a monoterpene alcohol used as a fragrance material, has been identified as a novel TRPA1 antagonist with a less odor and an analgesic effect against sensory irritation caused by high concentrations of menthol.

In our study, several ingredients formulated as anti-bacterial agents or humectants in cosmetics or drugs for external use, caused TRP channel activation. Among the anti-bacterial agents, methyl, ethyl, propyl, and butyl parabens activated TRPA1, but not other TRP channels expressed in sensory neurons, whereas phenoxy ethanol and sodium benzoate did not activate TRPA1. These differences are not dependent on their toxicity to human cells [29]. However, alkyl-chain length dependence of TRPA1 activation by parabens seemed similar to the anti-bacterial properties (Fig. 1H) [8]. Therefore, further investigation about these anti-bacterial agents leads novel theory for formulation of anti-bacterial agents. In addition, ethanol and polyols caused [Ca^2+^]_i_ increases in the cells expressing hTRPV1 and hTRPA1 (Fig. 2 and Additional Fig. 2A). Therefore, analyses of the correlation between TRPV1 and TRPA1 with sensory irritation are necessary when examining the sensory irritation properties of possible chemicals. In contrast, glycerin caused significant but weak [Ca^2+^]_i_ increases at 500 mM or 1 M in cells expressing TRPV1 (Fig. 2C) while 1 M glycerin did not induce inward currents in the patch-clamp experiment (Fig. 2H). Therefore, analysis using the patch-clamp technique is needed for a detailed understanding of the ingredients against TRP channels.

Since activation of hTRPV1 or TRPA1 might lead sensory irritation by using these products, we tried to analyze correlation between TRPA1 activities and sensory irritation by anti-bacterial agents and correlation between TRPV1 activities and sensory irritation by ethanol or polyols, respectively (Fig. 3). These correlation analyses indicate that TRPA1 and TRPV1 are useful tools to estimate sensory irritation by the ingredients. However, these estimations could be limited in similar characteristics because of large differences in concentrations and skin penetrating property of the ingredients. In the future, potentials in sensory irritation by the ingredients can be estimated by their TRP channel-activating abilities, molecular structures and molecular weights.

Even in the situation where cosmetics with minimal sensory irritation have been developed these days, some users still refrain from using cosmetics because of the risk of sensory irritation. And there are many reports describing the results of in vivo tests in humans. However, the effectiveness of the in vitro tests has been recently recognized. Lilja et al. reported that TRPV1 is a useful tool for detection of eye irritation with shampoo [30]. In the epidermis, sensory nerve endings express several TRP channels including TRPA1 and TRPV1, and they are important for nociception [11], [12], [13], [14], [15], [16], [17], [18]. Several cosmetic ingredients induce sensory irritation via TRPA1 or TRPV1 activation [19], [20], [21], [22]. And we showed that high concentrations of ethanol, DPG, or 1,3-BG cause [Ca^2+^]_i_ increases in the cells expressing hTRPV1, hTRPA1, or TRPV2 (Fig. 1, Fig. 2 and Additional Fig. 1, Fig. 2). This trend will more move forward in the near future. Participants tended to report a burning sensation in sensory irritation tests with ethanol after detergent treatment [32], which is consistent with the fact that TRPV1 is working as a heat sensor. And the role of TRPV2 in sensory irritation remains controversial [11], [31]. Although given these circumstances that TRPV1, TRPA1 and TRPV2 have to be considered as sensors of irritants, we want to propose that TRPA1 responding various cosmetic ingredients is the most important receptor against cosmetic ingredients for external use, based on the data shown in this study. Then, we decided to focus on TRPA1 as the target sensor to inhibit sensory irritation against several irritants formulated in cosmetics.

Arbanol inhibited TRPA1 activation caused by menthol, preservatives, or polyalcohols at a single cell level (Fig. 5). The transient enhancement of the hTRPA1 currents upon arbanol washout observed in our study might result from a bimodal action of the compound as observed for 1,8-cineole, borneol, 2-methylisoborneol, and fenchyl alcohol [23], [24]. On the other hand, arbanol has less odor or low volatility compared to previously reported TRPA1 inhibitors in vivo (Fig. 6). This might be a reason why this ingredient is used to arrange fragrance scent by controlling volatility of other fragrance chemicals. The observations that hTRPA1 inhibition by arbanol was affected by the mutations of N855, a binding site of specific TRPA1 agonist HC-030031 within the linker region of TM4 and TM5 [27], [33], and S873 and T874, binding sites of menthol in TM5 [28] indicate the arbanol action on hTRPA1 protein. However, only partial reduction in hTRPA1-inhibitory ability of arbanol was observed in the mutants. Further experiments with several analogs of arbanol or camphor on hTRPA1 or hTRPA1 mutants may be necessary to identify effective ingredients.

Since skin cosmetics are used daily and remain on the skin for extended periods, sensory irritation can affect consumer’s desirability. Although pain sensation and inflammatory responses must be avoided, sensory irritation that is not accompanied by inflammation should be avoided as well. However sensory irritation is difficult to measure quantitatively. In this regard, TRP channels are useful in evaluating sensory irritation by anti-bacterial agents, ethanol, and polyols. As an analgesic ingredient on hTRPA1 activity, arbanol with low odor contributes to non-irritating cosmetics that can be used by global consumers with sensitive skin.

## Ethics Statement

This study was conducted at Mandom Corporation and Okazaki Institute for Integrative Bioscience. Ethical approval of experiments involving human skin were obtained in Mandom Corporation (No.101–7, 102–34).

## Author Contributions

F.F. and M.Ta. conducted the study; S.I., K.Y., M.N., Y.Sh., K.U., provided advice and assisted with experiments for sensory irritation; M.S., H.S., K.I., Y.Su., provided advice and assisted with experiments for TRPA1 inhibitors; T.H. provided advice for statistical analysis; F.F. and M.To. managed the overall study as co-corresponding authors. All authors contributed to the study.

## CRediT authorship contribution statement

**Kiyomi Yoshikawa:** Supervision. **Shizuka Iwashita:** Validation. **Makoto Tominaga:** Writing – review & editing, Writing – original draft, Funding acquisition, Conceptualization. **Yoshiro Suzuki:** Supervision. **Fumitaka Fujita:** Writing – review & editing, Writing – original draft, Data curation, Conceptualization. **Kunitoshi Uchida:** Supervision. **Kenya Ishida:** Supervision. **Takeshi Hara:** Validation, Supervision. **Yuko Shiomi:** Supervision. **Moriaki Nagamatsu:** Supervision. **Hironori Shimizu:** Supervision. **Maki Sawada:** Supervision. **Masayuki Takaishi:** Data curation, Conceptualization.

## Declaration of Competing Interest

The authors declare the following financial interests/personal relationships which may be considered as potential competing interests:Makoto Tominaga reports financial support was provided by Japan Society for the Promotion of Science. If there are other authors, they declare that they have no known competing financial interests or personal relationships that could have appeared to influence the work reported in this paper.
