# Supplementary material for: Relationship between sensory irritation on skin surface and TRP channel activation
Source: J Physiol Sci. 2026 Aug 27;76(3):100096. doi: 10.1016/j.jphyss.2026.100096 (PMC13571459; doi:10.1016/j.jphyss.2026.100096)
Supplement: Supplementary file 1 [file mmc1.docx]

**Supplement information**

**Additional Fig. 1.** Increased cytosolic calcium concentration by anti-bacterial agents in HEK293 cells expressing hTRPV1 (a) (Methyl; n=182, Ethyl; n=151, Propyl; n=162, Butyl paraben; n=102, phenoxy ethanol; n=215 and sodium benzoate; n=61), M8 (b) (Methyl; n=51, Ethyl; n=32, Propyl; n=72, Butyl paraben; n=37, phenoxy ethanol; n=68 and sodium benzoate; n=43), or V2 (c) (Methyl; n=138, Ethyl; n=99, Propyl; n=43, Butyl paraben; n=136, phenoxy ethanol; n=40 and sodium benzoate; n=45). ** p<0.01 vs 1 mM Methyl paraben.

**Additional Fig. 2.** Increased cytosolic calcium concentration by ethanol or polyols in HEK293 cells expressing hTRPA1 (a) Ethanol (200 mM; n=48, 500 mM; n=15, 1 M; n=24), DPG (200 mM; n=41, 500 mM; n=66, 1 M; n=27), 1,3-BG (200 mM; n=49, 500 mM; n=18, 1 M; n=70), PG (200 mM; n=77, 500 mM; n=104, 1 M; n=87), and Glycerin (200 mM; n=76, 500 mM; n=31, 1 M; n=39) , * p<0.05, ** p<0.01 vs 200 mM ethanol or polyols, M8 (b) (Ethanol; n=46, DPG; n=28, 1,3-BG; n=26, PG; n=27 and Glycerin; n=22) or V2 (c) (Ethanol; n=76, DPG; n=31, 1,3-BG; n=62, PG; n=166 and Glycerin; n=88), * p<0.05, ** p<0.01 vs Glycerin.

**Additional Fig. 3.** Activation of hTRPM8 by arbanol (a) A representative trace of hTRPM8 current induced by 1 mM arbanol. (b) Dose-dependent activation of hTRPM8 by arbanol (n=5-7). The half-maximal effective concentration (EC_50_) and Hill’s coefficient values are 0.25 mM and 2.64, respectively.
